# Sepsis outcomes in patients receiving statins prior to hospitalization for sepsis: comparison of in-hospital mortality rates between patients who received atorvastatin and those who received simvastatin

**DOI:** 10.1186/s13613-015-0049-9

**Published:** 2015-05-06

**Authors:** Daniel R Ouellette, Erics Espinoza Moscoso, Julio Pinto Corrales, Michael Peters

**Affiliations:** Pulmonary and Critical Care Medicine, Henry Ford Hospital, K-17, 2799 West Grand Blvd, 48202 Detroit, MI USA; Department of Pharmacy, Henry Ford Hospital, K-17, 2799 West Grand Blvd, 48202 Detroit, MI USA

**Keywords:** Sepsis, HMG-CoA reductase inhibitors, Atorvastatin, Critical illness, Mortality, Inflammation

## Abstract

**Background:**

The purpose of this study is to compare the in-hospital mortality rates between septic patients receiving statins and those that did not prior to developing sepsis. We compared subgroups receiving atorvastatin and simvastatin because these two drugs differ in their pharmacologic properties.

**Methods:**

This study was a retrospective analysis of patients selected from an institutional data base of patients hospitalized with sepsis. The study patients were drawn from a data base of 1,961 hospitalized patients with sepsis and included patients who met selection criteria and who were studied for HMG-CoA reductase inhibitor (statin) use both prior to and during hospitalization. The in-hospital mortality rates of patients receiving statins and those that did not prior to developing sepsis were compared. In-hospital mortality rates of patient subgroups receiving atorvastatin and simvastatin were also compared. A multivariable analysis was conducted with in-hospital mortality as the outcome variable and with multiple risk factors to include atorvastatin and simvastatin use.

**Results:**

The mortality rate for 359 patients receiving statins prior to hospitalization for sepsis was not significantly different than that for 1,302 patients who did not receive pre-hospital statins (26.5% versus 30.4%, *p* > 0.05). The mortality rate for 92 patients who had received atorvastatin prior to hospitalization was significantly less than that of 253 patients who received simvastatin (18.5% versus 30.0%, *p* = 0.032). The use of atorvastatin prior to sepsis was independently associated with lower in-hospital mortality in a multivariable analysis of sepsis risk factors (*p* = 0.021, OR = 0.455). Patients who received atorvastatin prior to hospitalization for sepsis and had statins continued in hospital had a very low mortality rate that was significantly less than that of those patients who never received statins (15.7% versus 30.8%, *p* = 0.007).

**Conclusions:**

Pre-hospital atorvastatin use was associated with improved in-hospital mortality in septic patients when compared with pre-hospital simvastatin use and was independently associated with an improved outcome when compared to other sepsis risk factors. The effect of statins in patients with sepsis may be different for individual statins.

## Background

Sepsis and septic shock are formidable medical problems that challenge physicians caring for critically ill patients. Today, sepsis is one of the leading causes of morbidity and mortality with thousands of persons suffering from these conditions on a daily basis [1-4]. It has long been recognized that early and aggressive antibiotic treatment reduces mortality from sepsis [5]. Recent advances in resuscitation strategy and methods have also led to improved sepsis outcomes [6]. These advances have been embraced by the medical community and incorporated into guidelines and policy statements [7]. Despite these accomplishments, sepsis mortality remains high, with rates between 20% and 30% [8].

Sepsis is characterized by a complex, pleiotropic inflammatory response [8]. Amelioration of the inflammatory cascade in sepsis might be expected to have an impact on the clinical course and outcomes of the patient with sepsis. However, previous efforts to design treatment strategies to modify or disrupt the inflammatory cascade in the septic patient have been largely unsuccessful [9]. Statins are agents which have been observed to have important anti-inflammatory effects and to modulate the immune system response in a variety of ways during sepsis [10]. There has been speculation that the administration of statins may alter the inflammatory response to infection, suggesting that they may represent a potentially important adjunct to therapy [11]. Data suggests that critically ill patients may benefit from statins, and observational and retrospective studies have suggested that patients taking statins prior to the development of sepsis may have improved sepsis outcomes [12-14]. However, a recent large, prospective, multicenter study in patients with acute respiratory distress syndrome (ARDS) due to sepsis failed to demonstrate improved outcomes following the administration of rosuvastatin [15].

Though often considered together as a group, individual statins have unique biological properties. Statins are lipophilic to varying degrees, which may alter their individual effects. In addition, statins have recently been shown to have differential antibacterial properties *in vitro*, with atorvastatin having more prominent antibacterial effects than other statins [16]. We postulated that individual statins may have an agent-specific effect on outcomes in patients with sepsis and septic shock. We conducted a retrospective review of an institutional data base of patients with severe sepsis. Most patients in our institution who had received statins prior to hospitalization for sepsis had received either atorvastatin or simvastatin. We therefore compared the inpatient mortality rate of patients who had received atorvastatin to those who had received simvastatin.

## Methods

### Study design and enrollment

All patients at our institution who developed sepsis after January, 2005, had medical data entered into an institutional sepsis quality improvement data base. Patients admitted to the intensive care units with a diagnosis of sepsis, as well as those who developed sepsis during the course of their hospital stay for another diagnosis, were included in the data base. Patients with severe sepsis at our institution were transferred to the intensive care units and managed in this venue. We retrospectively reviewed data that was prospectively collected between January 1, 2005, and June 30, 2010. We additionally examined data from the institutional electronic medical record (EMR) and the hospital pharmacy data base for all patients enrolled in the sepsis data base. The study was performed at a quaternary health care system located in the Midwest United States. The study protocol was approved as an exempt protocol by the local institutional review board (IRB) project number 6870. The need for informed consent was waived.

Patient episodes were identified as those patients entered into the sepsis data base where the diagnosis of sepsis was confirmed by a retrospective review of the EMR. If a potential case subject had multiple admissions entered into the data base, only the first admission was considered for study purposes. We reviewed the EMR and the hospital system pharmacy data base to identify if the patient had a record of the use of statins prior to developing sepsis and whether they received statins during their hospitalization for sepsis. We recorded which statin each patient was receiving prior to and during hospitalization. We did not have specific information concerning the pre-hospital duration of treatment with statins. Due to the retrospective nature of the study, we were not able to assess pre-hospital compliance with prescribed statin therapy. The EMR was reviewed to identify the source of sepsis for each patient. We collected data from the EMR for each patient concerning comorbid conditions. The information for each study subject was entered into a research data base without patient identifiers for analysis.

### Definitions

*Sepsis* was defined as being present if a patient manifested at least two of four systemic inflammatory response syndrome criteria and had documented evidence of infection [17]. *Time 0* was defined as the point in time when the sepsis bundle was initiated for each study subject. *Intubation* was defined as endotracheal intubation and mechanical ventilation within 24 h of time 0. *In-hospital mortality* was defined as death from any cause during the hospitalization prior to discharge. *Time to antibiotics* was defined as the elapsed time in minutes from the initial presentation of sepsis to the initial administration of antibiotics.

We performed all statistical analysis using SPSS software version 18 with a logistical regression add-on package version 20 (IBM, Armonk, NY, USA) and considered *p* < 0.05 to be statistically significant unless otherwise specified. We used chi-squared tests for univariate analysis of dichotomous variables and two-sample *t* tests or Mann-Whitney *U* tests as appropriate for univariate analysis of continuous variables. Adjustments were not made for multiple comparisons. Where mean variables are listed, such data includes the standard deviation (mean ± standard deviation).

We developed a model to identify risk factors for mortality by multivariable logistic regression analysis in the subject populations. Potential risk factors were identified from a univariable analysis of each of the available variables using mortality as the dependent variable. Variables were selected for analysis if they were significantly associated with mortality (*p* < 0.05) and if at least 80% of the case and control subjects had data available for the variable.

## Results

**Figure 1:**
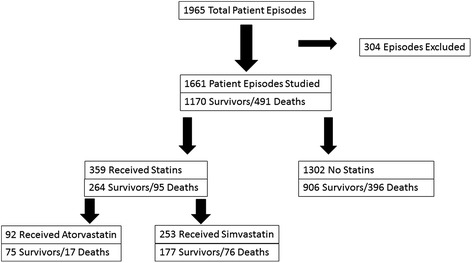
Organizational flowchart of patient episodes categorized by pre-hospital statin use and in-hospital mortality.

Clinical data were collected in a data base of patients with sepsis for 1,965 patient episodes of sepsis between January 1, 2005, and December 31, 2010. From these patient episodes, we selected 1,661 patient episodes of sepsis for study based upon the criteria described in the ‘Methods’ section (Figure 1). Exclusions included subsequent patient episodes after the first in patients with multiple sepsis events (the majority) and those where demographic data was incomplete. Of these patients, 58% were transferred to the ICU from the emergency department, 12% were transferred to the ICU from a general medical or surgical ward, and 29% were active patients in the ICU when sepsis was diagnosed. Patients received initiation of sepsis care as soon as sepsis was identified, regardless of the venue of care. Of the 1,661 patient episodes investigated, 1,170 resulted in survival to hospital discharge while 491 led to in-hospital death from all causes, providing an overall in-hospital mortality rate of 29.6% for the investigated population (Figure 1). The mean age (±standard deviation) for the population was 63 (±17) years, and the mean APACHE II score (±standard deviation) was 19 (±7). Male patients (53%) outnumbered female patients in our population. Overall, 44.4% of patients received vasoactive agents to support blood pressure, and 48.4% of patients were intubated and received mechanical ventilation within the first 24 h of sepsis. The primary sources of infection are listed in Table 1.

**Table 1:** **Sources of infection**

**Source**	**Number**	**Percentage of total (%)**
Respiratory	511	30.8
Urologic	263	15.8
Abdominal	217	13.1
Skin	126	7.6
Catheter	51	3.1
Blood	32	1.9
Heart	22	1.3
CNS	19	1.1
Bone and joint	3	0.2
Obstetrical	2	0.1
Sinus	2	0.1
Multiple	110	6.6
Unknown	303	18.2

**Figure 2:**
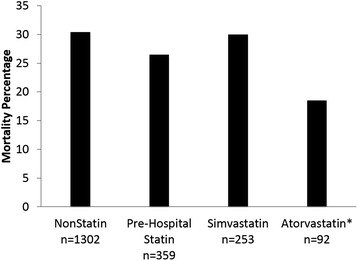
Comparison of in-hospital mortality between patient cohorts. *Pre-hospital atorvastatin vs pre-hospital simvastatin, *p* value = 0.032 (chi-square); pre-hospital atorvastatin vs no pre-hospital statin, *p* = 0.015 (chi-square).

There were 359 patients who received statins prior to hospitalization for sepsis. The in-hospital mortality rate for patients who received pre-hospital statins was not significantly different from the mortality rate for those patients not receiving pre-hospital statins (26.5% versus 30.4%, *p* = 0.146, Figure 2). We chose to compare the group of patients receiving pre-hospital atorvastatin to those receiving simvastatin. The demographic and clinical characteristics of these two groups were very similar (Table 2). Among the patients who received pre-hospital statins, 92 patients received atorvastatin, while 253 received simvastatin (Figure 1). Seventeen patients receiving pre-hospital atorvastatin died (18.5%), compared with 76 in the simvastatin group (30.0%), a difference which achieved statistical significance (*p* = 0.032, Figure 2). The atorvastatin group also had a significantly lower mortality rate than did those patients not receiving pre-hospital statins (*n* = 1,302, mortality rate = 30.4%, *p* = 0.015). We had dosing information available for all patients receiving pre-hospital simvastatin and atorvastatin. The mortality difference between groups of patients receiving different doses of simvastatin, or between these groups and the population of patients who did not receive pre-hospital statins, did not achieve significance. For atorvastatin, 5 of the 13 patients prescribed 10 mg of atorvastatin daily died, providing a mortality rate of 38.5%, which compares unfavorably with that of the 79 patients receiving higher doses of atorvastatin (15.2%, *p* = 0.045).

**Table 2:** **Comparisons between groups defined by pre-hospital statin use**

**Variable**	**Atorvastatin**	**Simvastatin**	Significance*	**No statins**	**Significance** ^**#**^
Number of patients	92	253		1,302	
Mean age (years)	67 ± 15	69 ± 13	*p* = 0.365^a^	61 ± 17	*p = 0.001* ^a^
Gender (male/female)	40/52	130/123	*p* = 0.194^b^	700/602	*p* = 0.056^b^
Mean temperature, time 0 (°C)	37.3 ± 1.5	37.2 ± 1.6	*p* = 0.766^a^	37.1 ± 1.5	*p* = 0.436^a^
Mean heart rate, time 0, (beats per min)	106 ± 22	107 ± 25	*p* = 0.860^a^	112 ± 25	*p = 0.033*a
Mean respiratory rate, time 0 (breaths per min)	24 ± 8	24 ± 9	*p* = 0.512^a^	25 ± 9	*p* = 0.351^a^
Mean arterial pressure, time 0 (mmHg)	76 ± 22	75 ± 22	*p* = 0.797^a^	76 ± 22	*p* = 0.974^a^
Mean arterial pH, time 0	7.34 ± 0.13	7.36 ± 0.12	*p* = 0.169^a^	7.35 ± 0.13	*p* = 0.577^a^
Mean creatinine, time 0 (mg/dL)	3.5 ± 3.0	3.1 ± 2.6	*p* = 0.357^c^	2.7 ± 2.9	*p = 0.003* ^c^
Mean leukocyte count, time 0 (K/μL)	14.7 ± 9.3	16.5 ± 12.7	*p* = 0.225^a^	16.1 ± 17.0	*p* = 0.459^a^
Mean hematocrit, time 0 (%)	32.6 ± 6.8	32.3 ± 6.5	*p* = 0.688^a^	32.9 ± 15.1	*p* = 0.860^a^
Mean platelet count, time 0 (K/μL)	242 ± 124	238 ± 130	*p* = 0.813^a^	230 ± 143	*p* = 0.431^a^
Mean bilirubin, time 0 (mg/dL)	1.2 ± 1.8	1.1 ± 2.1	*p* = 0.755^a^	1.6 ± 3.1	*p* = 0.221^a^
Mean lactate (mmol/L)	3.2 ± 2.8	3.6 ± 3.2	*p* = 0.454^c^	4.0 ± 3.4	*p = 0.011* ^c^
Mean APACHE II, time 0	18.8 ± 6.5	19.4 ± 7.1	*p* = 0.484^a^	18.6 ± 7.4	*p* = 0.833^a^
Time to antibiotics (min)	190 ± 152	166 ± 155	*p* = 0.300^a^	195 ± 226	*p* = 0.862^a^
Mean fluid volume administered, first 24 h (L)	4.542 ± 3.166	5.402 ± 3.348	*p = 0.036* ^a^	4.955 ± 4.477	*p* = 0.393^a^
Mean first CVP value (cm H_2_O)	12 ± 6	11 ± 6	*p* = 0.361^a^	10 ± 6	*p* = 0.123^a^
Median glucose, first 24 h (mg/dL)	142 ± 82	143 ± 56	*p* = 0.962^a^	134 ± 67	*p* = 0.226^a^
Mixed venous oxygen saturation (%)	71 ± 12	70 ± 15	*p* = 0.737^a^	70 ± 16	*p* = 0.753^a^
Use of vasopressors, number (%)	41 (44.6%)	115 (45.5%)	*p* = 0.883^b^	576 (44.2%)	*p* = 0.952^b^
Intubation, first 24 h, number (%)	37 (40.2%)	118 (46.6%)	*p* = 0.289^b^	641 (49.2%)	*p* = 0.095^b^
Dialysis, number (%)	7 (7.6%)	24 (9.5%)	*p* = 0.590^b^	48 (3.7%)	*p* = 0.062^b^
Abdominal source, number (%)	13 (14.1%)	29 (11.5%)	*p* = 0.503^b^	172 (13.2%)	*p* = 0.823^b^
Lung source (%)	27 (29.3%)	83 (32.8%)	*p* = 0.542^b^	396 (30.4%)	*p* = 0.314^b^
Urologic source (%)	16 (17.4%)	52 (20.6%)	*p* = 0.514^b^	192 (14.7%)	*p* = 0.872^b^
History of neoplasia (%)	16 (17.4%)	67 (26.5%)	*p* = 0.081^b^	334 (25.7%)	*p* = 0.077^b^
History of cardiac disease (%)	72 (78.3%)	196 (77.4%)	*p* = 0.876^b^	690 (53.0%)	*p < 0.0001* ^b^
History of chronic kidney disease (%)	51 (55.4%)	131 (51.8%)	*p* = 0.547^b^	410 (31.5%)	*p < 0.0001* ^b^
History of liver disease (%)	9 (9.8%)	20 (7.9%)	*p* = 0.578^b^	252 (19.4%)	*p = 0.023* ^b^
Mortality (%)	17 (18.5%)	76 (30.0%)	*p = 0.032* ^b^	396 (30.4%)	*p = 0.015* ^b^

*Comparison between patients receiving atorvastatin or simvastatin prior to hospitalization; ^#^comparison between patients receiving atorvastatin or no statins prior to hospitalization. ^a^Student’s *t* test; ^b^chi-square test; ^c^Mann-Whitney *U* test. Values achieving significance are italicized.

In addition to those patients noted who had received atorvastatin or simvastatin, six received lovastatin, five received rosuvastatin, and two received pravastatin. One patient had reported statin use, but the specific agent used was not determined. Two patients died who had received lovastatin, one patient died of the two receiving pravastatin, and no patients receiving rosuvastatin died.

In order to determine if the use of atorvastatin or simvastatin in the pre-hospital setting was an independent risk factor associated with mortality, we performed first a univariable followed by a multivariable analysis of potential risk factors, using mortality as the dependent variable. All 1,661 patient episodes were included in the analysis. We chose a total of 32 potential risk factors to study by univariable analysis, including all the variables listed in Table 2 plus three additional risk factors: pre-hospital atorvastatin use, pre-hospital simvastatin use, and pre-hospital statin use. Pre-hospital atorvastatin was significantly associated with reduced mortality (*p* = 0.018), whereas pre-hospital simvastatin was not (*p* = 0.856) in the univariable analysis. Risk factors that were significantly associated with mortality (*p* < 0.05) in the univariable analysis and for which data was available for at least 80% of patient episodes were included in the multivariable analysis. The results of the multivariable analysis are presented in Table 3. We determined that pre-hospital atorvastatin use was significantly and independently associated with improved mortality in septic patients (*p* = 0.021, OR = 0.455).

**Table 3:** **Multivariable analysis of the association of clinical risk factors with mortality**

**Risk factor**	**Significance**	**Odds ratio**
Platelet count, time 0	*p* = 0.001	0.998
Lactate value	*p* < 0.0001	1.083
APACHE II	*p* < 0.0001	1.038
Glucose level	*p* = 0.091	0.998
Male gender	*p* = 0.073	1.273
Vasopressor use	*p* < 0.0001	1.678
Mechanical ventilation	*p* < 0.0001	2.114
Urologic source of infection	*p* < 0.0001	0.434
Oncologic past history	*p* = 0.045	1.355
Atorvastatin use prior to sepsis	*p* = 0.021	0.455
Age	*p* = 0.003	1.013
Temperature, time 0	*p* = 0.091	0.928
Mean arterial pressure, time 0	*p* = 0.459	0.998
Leukocyte count, time 0	*p* = 0.071	1.010
Hematocrit, time 0	*p* < 0.0001	0.956
Arterial pH	*p* = 0.342	1.796

Of the 1,302 patients that did not receive pre-hospital statins, 61 patients received statins after hospitalization. The mortality rate of the patients receiving statins in hospital but not before hospitalization was less than that of patients who never received statins, but this difference did not reach significance (23% versus 30.8% respectively, *p* = 0.222). Of the patients who received statins only in hospital, twenty-nine of these patients received atorvastatin with eight such patients expiring (mortality rate = 27.6%).

Of the 359 patients who received statins prior to hospitalization, 267 had such therapy continued in hospital. The patients who received statins both prior to hospitalization and during hospitalization had an in-hospital mortality rate of 24.7% compared with a rate of 31.5% for patients who had received statins prior to hospitalization but had this therapy discontinued (not significant, *p* = 0.209).

Of the 92 patients who received atorvastatin prior to hospitalization, 70 patients had statins continued in hospital (9 patients received simvastatin rather than atorvastatin in-hospital). These 70 patients had a mortality rate of 15.7%, which was significantly different from the population of patients that had never received statins (*p* = 0.007) and was less than but not significantly different from the mortality rate of the 22 patients that had received atorvastatin in the pre-hospital setting and then who did not receive statins in hospital (27.2%, *p* = 0.223). There were 60 patients who received only atorvastatin both prior to and during hospitalization; 9 of these patients died, providing a mortality rate of 15%.

## Discussion

The most important finding in our study is that pre-hospital atorvastatin use was significantly associated with reduced mortality during a hospitalization for sepsis when compared to pre-hospital simvastatin use. Pre-hospital administration of atorvastatin was an independent factor associated with improved mortality in our septic population despite the fact that statins as a class were not associated with improved mortality when administered prior to hospitalization. Patients who received atorvastatin prior to a hospitalization for sepsis, and had statins continued in hospital, had a very low mortality rate that was significantly different than patients who never received statins and less than but not significantly different than patients who only received atorvastatin in the pre-hospital setting. Some, but not all, prior studies suggest that administration of statins prior to the development of sepsis may improve sepsis outcomes [12,18,19]. Little work has been done in this regard concerning individual statin agents.

Although the focus of our investigations was statin use in septic patients, we also made observations concerning other sepsis mortality risk factors. Risk factors in our patients significantly associated with increased mortality, and those which have been observed by other investigators to be associated with increased mortality during severe infection include thrombocytopenia [20], high lactate levels [21], APACHE II score [22,23], vasopressor use [23,24], mechanical ventilation [23,25], a past history of cancer [26], age [23], and anemia [27]. We noted that having a urologic source of sepsis, as opposed to other sources of infection, was associated with a protective effect against mortality, which has also been previously observed [28].

Recent studies have tempered the early enthusiasm for the role of statins in the treatment of sepsis [29]. Janda and coworkers performed a systemic review and meta-analysis of 20 studies, including prospective, retrospective, observational, and cohort studies [19]. A protective effect of statins compared with placebo was demonstrated for a variety of infectious outcomes, but the analysis was very limited by the quality of the studies. Pasin and colleagues assessed five prospective, placebo-controlled studies involving statins in a meta-analysis and found that there was no evidence of a difference in mortality or hospital stay [30]. Wan and associates evaluated both prospective, controlled studies and observational studies [31]. Among five prospective, randomized, controlled studies, there was no demonstrable change in in-hospital or 28-day mortality. However, among 27 observational studies, there was a significant decrease in mortality noted. A recent large, multicenter, prospective, randomized, and controlled study comparing rosuvastatin to placebo in patients with ARDS due to sepsis failed to demonstrate a mortality benefit for rosuvastatin use [15]. In our study, we found that pre-hospital statin use generally did not change outcomes, though atorvastatin was associated with reduced mortality.

Statins are commonly used as lipid-lowering agents. Although often considered as a class, statins vary between agents in terms of their chemical structure and properties and have different pharmacodynamics [32]. While some statins have been derived from fungal metabolites, others, such as atorvastatin, are completely synthetic. Statins vary considerably in terms of their lipophilicity, first-pass metabolism, half-life, and bioavailability. Metabolism of statins varies among agents, and activity at the active site of the enzyme HMG-CoA reductase is subtly different for different statins. Because of these chemical and pharmacological differences between statins, different biological effects might be anticipated.

Statins have been postulated to have an effect on sepsis outcomes that is mediated by their anti-inflammatory effects [10,11]. Recent evidence has suggested that statins also may have direct antimicrobial properties. Masadeh and colleagues studied the *in vitro* antimicrobial effect of statins on 16 common bacterial strains, including both gram-positive and gram-negative bacteria, finding evidence that different statins had varying antimicrobial effects [16]. While both atorvastatin and simvastatin were more potent than rosuvastatin with respect to many gram-positive agents, selected gram-negative organisms were more sensitive to atorvastatin than either simvastatin or rosuvastatin. However, it should be noted that the minimum inhibitory concentrations for atorvastatin, in particular, in the work by Masadeh, exceed by at least 100-fold the maximum serum concentration for this agent seen in human subjects with standard dosing [33]. This fact limits the extrapolation of Masadeh’s *in vitro* data to a clinical effect in sepsis.

Individual statins have not been directly compared with respect to clinical outcomes from infection. We assessed the 27 observational studies highlighted in the comprehensive review by Wan [31], finding that 15 did not distinguish between the types of statins used, and 1 study reported simvastatin use. Of the 11 studies reporting and specifying the individual statins used, none directly compared clinical outcomes between statins. Prospective studies to date have compared individual statins to placebo, but not to other statins. Our work represents the first comparison of in-hospital mortality rates in septic patients treated prior to hospitalization with two different statins, with the finding of improved mortality associated with atorvastatin compared to simvastatin.

Statins have beneficial effects on lipid profiles and cardiovascular outcomes and are generally well tolerated in stable outpatients. Despite the possible beneficial effects noted in this work in patients with sepsis, statins may have adverse effects which could complicate their use, especially in the critically ill patient. Rhabdomyolysis and myopathy are known complications of statin use. Statins are metabolized by the cytochrome P450 system in the liver, and liver disease, a common occurrence in the critically ill, affects the metabolism of these agents and increases the risk of muscle disease [32]. Food intake has a variable effect on statin bioavailability, which may have important consequences in the critically ill patient that is unable to receive oral nutrition [32]. Statins are highly protein bound, a fact that may well be important in the critically ill patient [32]. Interruption of chronic outpatient therapy in a critically ill patient could lead to augmentation of cardiovascular risk [14]. We did not have specific information concerning potential adverse effects from statins, including cause-specific mortality and cardiovascular morbidity, which limits our study in recommendations concerning statin utility in sepsis.

Our study has a number of important limitations. Our work is from a single center and is retrospective and observational. We did not have information about pre-hospital duration of statin therapy, nor did we have information concerning patient compliance with prescribed pre-hospital statin treatments. While most of our patients had health insurance, the lack of coverage for some patients creates the possibility of treatment bias along demographic lines within our population. In addition, insurance policies provide economic advantages for the use of specific agents within a class, a fact which clearly has implications for our study. Given the volatile and changing insurance environment within the United States during the period in question, we have no ability in our retrospective study to completely account for these effects. We performed multiple comparisons in analyzing our data without adjustment, increasing the likelihood of false-positive associations. However, we note that there was little difference between the group of patients receiving atorvastatin and those receiving simvastatin, except for the total volume of fluid received during resuscitation. While resuscitation with intravenous fluid and other agents to defined clinical goals is an important element of sepsis management, we have previously demonstrated in our patients that the total amount of fluid administered in the first 24 h of the initial resuscitation is not an independent risk factor for adverse clinical outcomes [34].

## Conclusions

Pre-hospital atorvastatin use by patients developing sepsis was associated with reduced in-hospital mortality when compared to the use of pre-hospital simvastatin. Pre-hospital atorvastatin was independently associated with an improved mortality rate when controlling for other sepsis risk factors in a population of septic patients. We postulate that atorvastatin may have unique biochemical, pharmacodynamic, and antimicrobial effects compared with other statins that may account for the observed amelioration in mortality rates. Further study of the effects of individual statin agents in patients with sepsis is needed to confirm this hypothesis.

## Abbreviations

APACHE II: Acute Physiology and Chronic Health Evaluation II
ARDS: Acute respiratory distress syndrome
EMR: Electronic medical record
HMG-CoA: 3-hydroxy-3-methylglutaryl-coenzyme A
IRB: Institutional review board
